# First human study in treatment of unresectable liver metastases from colorectal cancer with irinotecan-loaded beads (DEBIRI)

**DOI:** 10.3892/ijo.2012.1572

**Published:** 2012-07-25

**Authors:** K. EICHLER, S. ZANGOS, M.G. MACK, R. HAMMERSTINGL, T. GRUBER-ROUH, C. GALLUS, T.J. VOGL

**Affiliations:** Department of Diagnostic and Interventional Radiology, University Hospital Frankfurt, Johann Wolfgang Goethe-University, D-60590 Frankfurt, Germany

**Keywords:** intraarterial therapy, liver metastases, colorectal cancer, beads

## Abstract

The objective of this pilot clinical study was to assess the safety, technical feasibility, pharmacokinetic (PK) profile and tumour response of DC Bead™ with irinotecan (DEBIRI™) delivered by intra-arterial embolisation for the treatment of metastatic colorectal cancer. Eleven patients with unresectable liver metastases from CRC, tumour burden <30% of liver volume, adequate haematological, liver and renal function, performance status of <2 were included in this study. Patients received up to 4 sessions of TACE with DEBIRI at 3-week intervals. Feasibility of the procedure, safety and tumour response were assessed after each cycle. PK was measured after the first cycle. Patients were followed up to 24 weeks. Only mild to moderate adverse events were observed. DEBIRI is a technically feasibile procedure; no technical complications were observed. Average C_max_ for irinotecan and SN-38 was 194 ng/ml and 16.7 ng/ml, respectively, with average t_½_ of 4.6 h and 12.4 h following administration of DEBIRI. Best overall response during the study showed disease control in 9 patients (2 patients with partial response and 7 with stable disease, overall response rate of 18%). Our study shows that transarterial chemoembolisation with irinotecan-loaded DC beads (DEBIRI) is safe, technically feasible and effective with a good PK profile.

## Introduction

Despite the existence of excellent screening and preventive strategies, colorectal carcinoma remains a major public health problem in western countries. The American Cancer Society estimates there will be 142,570 new cases diagnosed in 2010 and 51,370 people will die of the disease. In Germany, the incidence was about 70,404 in 2010 and 27,582 people died of colorectal cancer (2). Hepatic metastasis of colorectal cancer is common and by the time of diagnosis, 25% of colon cancers and 50%–70% of cancers of the rectum will have extended through the bowel wall metastasising to lymph nodes in 50%–60% (3). The most common site of extra-lymphatic involvement is the liver, with the lungs as the most frequently affected extra-abdominal organ. Patients with metastatic colorectal tumours frequently die of hepatic failure due to liver metastases. Therefore, treatment of liver metastases may prolong survival, even in the presence of extra-hepatic disease.

Treatment options for patients with liver metastatic colorectal cancer are limited and clinical outcome is generally poor. Surgical resection in selected patients can achieve 30–70% 5-year survival (4–6). However, only 20–30% of patients with colorectal liver metastases are suitable for resection at initial presentation (7,8) and 70–80% of patients will develop recurrence within 5 years (9).

Untreated colorectal metastases to the liver have a poor prognosis and are associated with a median survival of 5–10 months (10). Systemic chemotherapy can palliate symptoms and improve survival. 5-Fluorouracil (5-FU)-based chemotherapy has been the cornerstone of treatment of unresectable CRC for more than 40 years, and new drugs such as irinotecan, oxaliplatin, cetuximab and bevacizumab have recently broadened the options for treatment. With the introduction of these novel agents, median survival has increased significantly from 6 months to over 20 months (11).

In addition to systemic chemotherapy, current therapies of unresectable liver lesions include hepatic arterial infusion of chemotherapeutic drugs, transarterial chemoembolisation, radiofrequency ablation, cryotherapy, laser-induced thermotherapy (LITT) and yttrium-90 radioembolization (12–16).

Recent reports on the use of irinotecan loaded DC beads (DEBIRI) in transarterial chemoembolisation (TACE) therapy for metastatic colorectal cancer show promising results (17,18). These early reports suggest DEBIRI as a safe and effective treatment for patients with liver predominant unresectable metastases from colorectal cancer. This study was the first clinical evaluation in the development of DEBIRI. Critical features of this study relate to safety (adverse events), technical feasibility, PK, profile and efficacy (tumour response, time to progression and necrosis) which were considered pivotal in the assessment of DEBIRI as a therapy for unresectable metastases from colorectal cancer.

## Materials and methods

This prospective, pilot, single-arm study, approved by an independent ethics committee, was conducted in our institution from January 2006 to April 2008. Thirty patients were planned to be enrolled, but the study was closed after 11 patients were included due to the slow recruitment rate.

Colorectal cancer stage IV (Dukes’ D, TNM: TX, NX, M1) patients were eligible for enrolment if they gave written informed consent, were at least 18-year-old, of any race or sex, had their primary tumour(s) successfully removed by surgery, had 1 to 8 measurable unresectable metastase(s) that were confined to the liver, with a tumour burden of no more than 30% of liver volume. In addition, patients were required to have an ECOG performance status score of ≤1, with a life expectancy of >6 months, and adequate liver, renal and hematologic function. Women of child bearing potential and fertile men were required to use effective contraception and prior therapy was permitted (with or without irinotecan) if the last chemotherapy or prior radiotherapy treatment was completed at least 4 weeks before enrolment. Exclusion to therapy included the presence of extrahepatic metastases, any contraindications to irinotecan, active bacterial, viral or fungal infection, presence of another concurrent malignancy, prior malignancy in the last 5 years (except adequately treated basal or squamous cell skin cancer or carcinoma *in situ* of the cervix) and any contraindication for hepatic embolisation procedures.

All patients had baseline MRI/CT to document the extent of liver disease prior to therapy. Patients were to receive TACE treatments with DEBIRI as monotherapy every 3 weeks (up to 4 treatments) and followed up for 6 months. Last chemotherapy or prior radiotherapy treatment had to be completed at least 4 weeks before study entry (first treatment).

The primary study endpoint of safety was measured by adverse events, serious adverse events, and physical and laboratory assessments. Secondary study endpoints of technical feasibility, pharmacokinetic profile (irinotecan and SN-38) and efficacy [tumour response and time to progression (TTP) measured by MRI/CT assessments according to RECIST at 3 and 6 months following the first DEBIRI treatment] were also measured. Where feasible, the percentage necrosis was calculated as total necrotic tumour volume divided by total treated tumour volume.

### Transarterial chemoembolisation with irinotecan loaded DC bead (DEBIRI)

DC Bead (Biocompatibles, Farnham, UK) comprise a range of modified polyvinylalcohol hydrogel microspheres that are biocompatible, hydrophilic, non-resorbable, and precisely calibrated. DC Bead is a CE marked drug delivery embolisation system and is indicated for loading irinotecan (DEBIRI) for embolisation of vessels supplying malignant colorectal cancer metastasised to the liver (mCRC) and is capable of eluting a local, controlled, sustained dose of irinotecan to hepatic metastases of colorectal cancer. DC Beads of 100–300 μm and 300–500 μm in size were loaded with irinotecan to dose of 50 mg/ml beads according to the instructions for use. After removing saline from the vials, 5 ml of 20 mg/ml irinotecan solution was added to each vial of DC Bead. At the time of our study, vials were stored for at least 4 h for loading; however, the recommended loading time is 2 h. At the end of the loading period the supernatant was discarded and beads mixed 50:50 with non-ionic contrast (Omnipaque^®^).

All patients were adequately pre-medicated according to standard hospital procedure for chemoembolisation and, where required, included prophylactic pain management, anti-emetics and corticoids using 100 mg Pethidin (Dolantin^®^), 3 mg Granisetron (Kevatril^®^) and 20 mg Fortecortin, 10 ml Mepivacain (Scandicain^®^) was also used for local anaesthesia subcutaneously. Additional pain medication and/or vasodilators were used at the investigator’s discretion.

A diagnostic angiogram was performed to evaluate the hepatic aterial supply and verify portal patency. The right or left hepatic artery was selected, depending on the location of the lesions to be treated. Once the vascular supply of the tumour was identified, a microcatheter was placed superselectively and distal to the cystic artery in close proximity to the lesions. Irinotecan loaded DC Bead was injected slowly until complete stasis of blood flow was achieved (embolisation endpoint). Up to 8 ml of loaded DC Bead (400 mg irinotecan) was to be delivered until the embolisation endpoint was achieved. This dose was considered safe for local delivery based on two preclinical studies in a porcine liver model (19) and is less than the recommended 3-weekly dose of irinotecan monotherapy (350 mg/m^2^). Infusion of the beads was stopped if the embolisation endpoint was achieved prior to delivery of the full dose. No additional embolic agent was used to achieve the embolisation endpoint. Following the procedure all patients were observed for vital signs, femoral access site, pain management, fluid hydration and discharged according to standard hospital procedure.

### Pharmacokinetics

Blood samples were taken prior to and at 5, 10, 15 and 30 min, 1, 2, 4, 6 and 24 h and on day 21 after the first DEBIRI treatment. Plasma levels of irinotecan and SN-38 were measured using an HPLC fluorescence method previously validated at CentraLabs Clinical Research (Huntington, UK). Peak plasma concentrations (C_max_), area under the curve (AUC) and plasma half-life (t_½_) were measured for irinotecan and its main active metabolite SN-38.

### Statistical consideration

This was a pilot phase I/II study intended to assess feasibility. No well-documented ‘gold-standard’ protocol for chemoembolisation of liver metastases that could serve as a control is recognised by the medical community. Furthermore, placebo-control in patients with metastatic cancer was considered unethical and ruled out. A single-arm, non-comparative study was thus the suitable design for this initial safety study. A planned enrolment of 30 patients was considered adequate for this assessment. Descriptive and quantitative statistics were used to summarise categorical variables and continuous variables. When relevant, the 95% confidence intervals (CI) were also reported. Spearman rank correlation was used to determine any relationship between dose and adverse events. Time to progression was calculated using Kaplan-Meier. Paired t-tests and non-parametric sign tests were performed on paired data continuous variables. The latter was used because of the small sample size of this study.

## Results

### Demographics

A total of 11 patients (8 males, 3 females) with a mean age of 64 years (range 45–85 years) were enrolled in the study. All patients had prior surgery to successfully remove their primary colorectal cancer. Eight patients had further treatment for their liver metastases including, laser-induced interstitial thermotherapy, radiofrequency ablation and radiotherapy. Mean sum of longest diameter in target lesions was 63±25 mm (range 19–102 mm) with percentage liver involvement of 8.2±2.6% (range 4–12%). Eight patients had information on prior chemotherapy (Table I).

**Figure 1:**
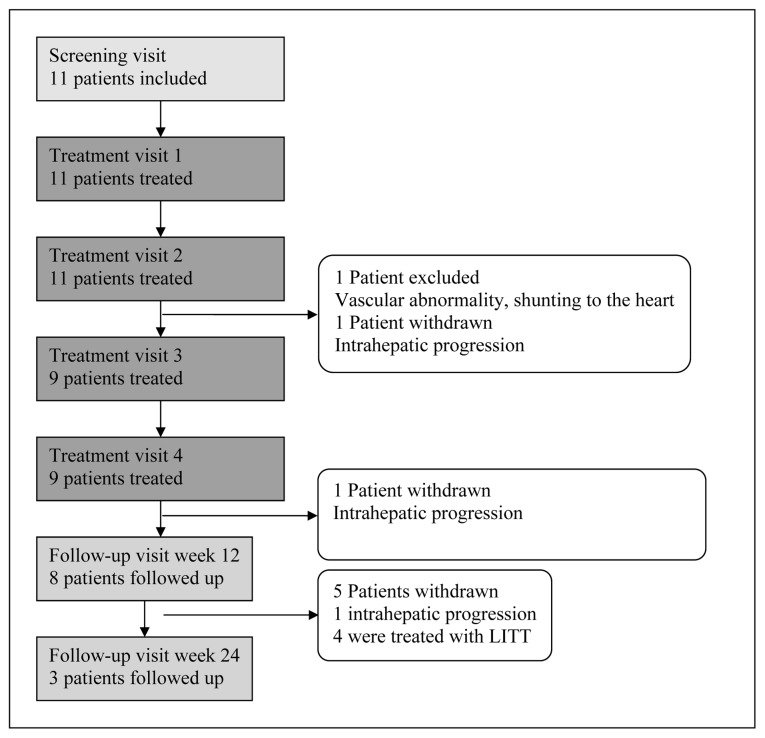
CONSORT overview of study participation.

Of the 11 patients enrolled, 9 patients successfully received 4 TACE treatments with DEBIRI. Two patients were withdrawn after the second DEBIRI treatment, one due to a vascular abnormality with shunting to the heart, the other due to intrahepatic progression (Fig. 1). The median follow-up period for all the patients was 83 days (range, 24–176 days). Three patients withdrew from the study due to intrahepatic progression whilst 4 patients were considered eligible for laser-induced interstitial thermotherapy (LITT) following DEBIRI (Fig. 1).

### Treatment

The pre-planned dose of irinotecan loaded DC Bead was up to 400 mg (8 ml beads) in each treatment. Final delivered dose varied in the patients and was dependant upon volume of beads delivered prior to the embolisation endpoint.

In all cases, the embolisation endpoint (stasis of the subsegmented artery) was achieved with less than 4 ml (200 mg irinotecan). In the 40 DEBIRI treatments performed, between 21–143 mg irinotecan was delivered in each treatment session and an average total dose of 293 mg (range 124–440 mg) per patient was achieved across all their individual treatments. Bead size of 100–300 μm and 300–500 μm were used throughout the treatments.

### Safety

Ten patients retained an ECOG status of ≤1 during the study. Nine of the 11 patients (81.8%) experienced a total of 41 adverse events during the study. Each adverse event (AE) was coded according Medical Dictionary for Regulatory Activities, MedDRA version 10.0 (MSSO 2007) (Table II). Gastrointestinal disorders were the most common AEs (63%) predominantly due to abdominal pain, nausea and vomiting. These are consistent with the expected post-embolisation syndrome observed in patients undergoing chemo-embolisation of the liver. The majority of AEs were mild (61%) with the remainder graded as moderate (39%). No irinotecan related toxicities such as delayed diarrhoea or neutropenia were observed.

No serious adverse events (SAEs) were reported during the study and no clinically significant changes from baseline values were observed in the laboratory findings. The Spearman’s rank correlation coefficient was calculated to examine whether an association existed between dose delivered and number of adverse events. The coefficients (rs =0.289 and 0.187 for total dose and mean dose, respectively) showed no evidence of an association between dose and total number of adverse events experienced.

### Technical feasibility

Forty DEBIRI treatments were successfully performed in these 11 patients. No technical complications were reported during the study demonstrating feasibility of DEBIRI in the treatment of patients with liver metastases from colorectal cancer.

### Pharmacokinetics (PK)

PK analyses were completed for 10 patients. Average dose of irinotecan delivered in PK patients was 86 mg. Peak plasma concentrations (C_max_), area under the curve (AUC) and plasma half-life (t_½_) results for irinotecan and its main active metabolite SN-38 are shown in Table III. C_max_ for irinotecan and SN-38 were 194 ng/ml and 16.7 ng/ml, respectively, following administration of DEBIRI. Average AUC values were 1,680 ng•h/ml and 281 ng•h/ml, respectively. t_½_ ranged from 1.6–7.2 h (mean 4.6 h) for irinotecan and 7.6–8.5 h (mean 12.4 h) for SN-38. Results for AUC and t_½_ should be treated with caution as data from the majority of patients did not meet the data acceptance criteria to calculate the slope of λ_z_ requiring a minimum of 3 terminal data points randomly distributed on a straight line with a regression coefficient of ≥0.95, the fraction of the variance accounted for was ≥0.90 and an interval at least 2-fold greater than t_½_.

### Efficacy

**Figure 2:**
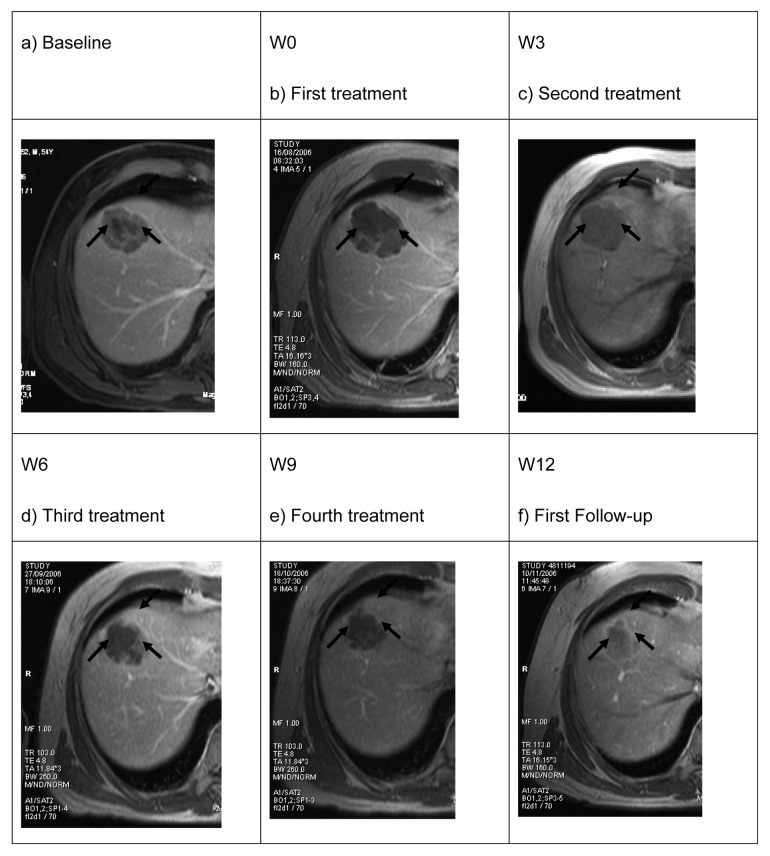
Fifty-four-year-old patient with a liver metastases in liver segment eight. After the first treatment devascularisation and necrosis in the lesion is observed, partial response.

Tumour response was assessed according to Response Evaluation Criteria in Solid Tumours (RECIST) (20). Up to 5 target lesions in the liver were followed and the sum of longest diameters used to assess tumour response. Stable disease was reported in 9 (81%) of patients after the first DEBIRI treatment and 7 (64%) remained stable by the third treatment (week 9) with one achieving partial response (Fig. 2).

By the end of the study, intrahepatic progression of the disease according to RECIST was reported in 7 patients (63%), a partial response to treatment in 2 patients (18%) and stable disease reported in 2 patients (18%) (Table IV). These four patients received the LITT treatment in the follow-up. Best overall response was defined as the best response from start of treatment over all follow up visits or until disease progression/recurrence. Best overall response during the study showed disease control in 9 patients (2 patients with partial response and 7 with stable disease).

It was planned to measure the extent of tumour necrosis following DEBIRI, however, we found that tumour necrosis was difficult to assess and only possible in 3 patients. No conclusions could be drawn from this assessment.

### Tumour markers

Carcinoembryonic antigen (CEA) and cancer/carbohydrate antigen 19-9 (CA 19-9) (21) were measured pre- and post-DEBIRI treatment. Nine (82%) patients had clinically significant levels of CEA at baseline that remained throughout the study. Three patients (27%) saw an average decrease of 71% (range 61 to 79%) in their CEA levels. When compared to overall treatment outcome (RECIST), an increase in CEA level from baseline to last measurement was associated with disease progression in 6 of the 7 (86%) patients. In the 3 patients with a decrease in CEA level compared to baseline values, a stable disease or partial responses was observed. A similar trend was also found in the CA 19-9 results.

### Time to progression (TTP)

**Figure 3:**
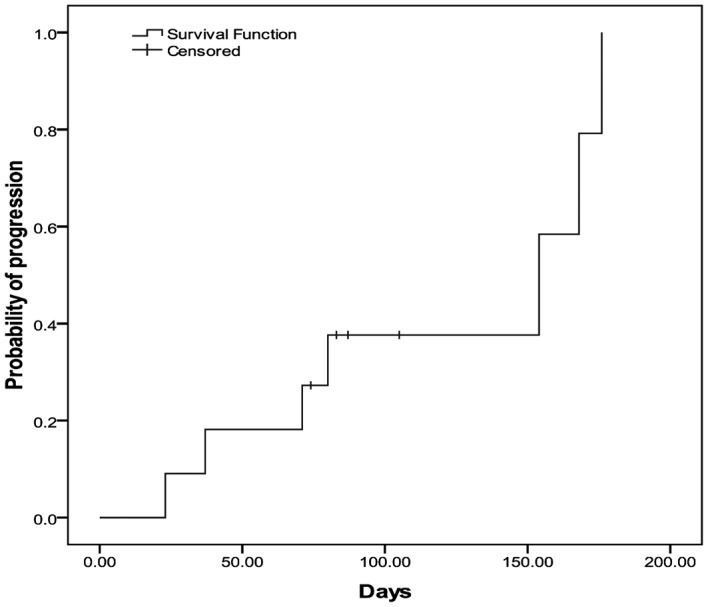
Probability of progression according to RECIST.

Probability of disease progression was determined using the Kaplan-Meier method. Based on responses assessed at the treatment and follow-up visits, median TTP from first treatment was 154 days (95% CI, 17–291 days) (Fig. 3).

## Discussion

The management of metastatic colorectal cancer is becoming far more complex and requires a multidisciplinary and collaborative approach in selecting the optimal treatment for patients with liver metastases of colorectal cancer. Despite advances in the development of new cytotoxics and targeted biologics for the treatment of hepatic metastases from colorectal cancer, there is still interest in liver directed, locoregional therapy to improve treatment response and potentially improve survival. Prognosis in these patients remains poor with 1- and 3-year median survival rates of 31% and 2% (5,22–24). With response rates in second-line systemic chemotherapeutic treatments between 4–22%, a more precise chemotherapeutic delivery system to maximize response rates and reduce side effects is a potential option in the treatment algorithm for hepatic metastases from colorectal cancer (25).

Transarterial chemoembolisation (TACE) is a locoregional therapy that is most widely used for the treatment of unresectable HCC (26–28) and involves the periodic injection of a chemotherapeutic agent, mixed with an embolic material, into selected branches of the hepatic arteries feeding a liver tumour. The expected advantage of TACE is that higher concentrations of the drug can be delivered to the tumour with decreased systemic exposure compared with systemic chemotherapy. We have learnt from hepatic artery infusion (HAI), that even tumours unresponsive to systemic therapy may benefit from the higher doses afforded by local delivery (29).

Early reports on the use of conventional TACE in the treatment of hepatic metastases from colorectal cancer were encouraging (30) and data from several reports have now emerged on effectiveness, response rates, side effects and overall survival. One of the largest series published evaluated the efficacy of TACE with respect to local control and survival (31). Patients (n=463) with liver metastases from colorectal cancer were treated with repeated TACE at 4-week intervals. In total, 2,441 chemoembolisations were performed with a mean of 5.3 sessions per patient. The chemotherapy consisted of mitomycin C with/without gemcitabin or mitomycin C with irinotecan and embolisation was performed with lipiodol and starch microspheres for vessel occlusion. Tumour response according to RECIST 3 months after the three TACE administrations was evaluated by MRI. Partial response was observed in 12% of patients, stable disease in 51% and progressive disease in 37%. The 1-year survival rate was 62%, but dropped to 38% by 2 years. Median survival time from first TACE treatment was 1.34 years (31).

In recent decades, many efforts have been made to improve the outcome of TACE by integrating new chemotherapeutic agents and providing a more accurate dosage of drug delivery to the liver for a more prolonged period. DC Bead is capable of loading irinotecan and release the drug in a sustained way in the liver after injection in the hepatic artery. Fiorentini *et al*(17) evaluated irinotecan loaded DC Beads (DEBIRI) in 20 patients with liver metastases from colorectal cancer in a palliative setting. A relevant response was observed in 16 out of 20 patients, with significant reduction of lesional contrast enhancement in all responding patients. The procedure was well tolerated by most patients with a median duration of hospitalization of 3 days (range 1–10 days). The most important adverse effect was abdominal pain, especially during injection of irinotecan loaded DC beads.

A randomised, phase III study (1), compared DEBIRI vs FOLFIRI in patients who failed 2/3 lines of systemic chemotherapy. At a median follow-up of 24 months, median overall survival was improved by 43% in the DEBIRI arm (690 days) compared with the FOLFIRI arm (482 days) with significantly better response and PFS in favour of DEBIRI (68%, 225 days vs 18%, 94 days, respectively). Acute toxicity (G3 pain, nausea, fever) was greater with DEBIRI and was observed in the context of post-embolisation syndrome, and were controlled with intravenous hydration, morphine and antibiotics. Late toxicity was significantly higher with FOLFIRI. Diarrhoea, asthenia, leucopenia, anaemia and G3 fever were substantially less with DEBIRI (2–20% vs 35–50%). This is the first study to report a clear survival benefit of DEBIRI over systemic chemotherapy with a reduction in overall cost. Martin *et al* published data from an open-label multi-center study where 55 patients with unresectable liver metastases from CRC underwent 99 DEBIRI treatments (32). Median length of hospital stay was 24 h. Median disease-free and overall survival from the time of first treatment was 247 days and 343 days, respectively. Six patients (10%) were downstaged from their original disease status.

In our pilot study, 11 patients with up to 8 hepatic lesions from CRC were treated with DEBIRI. This was the first clinical evaluation in the development of DEBIRI and aimed to evaluate the safety, technical feasibility, PK profile and efficacy of this new treatment.

The treatment showed an excellent safety profile with no serious adverse events reported. No clinically significant change in blood biochemistry or dose limiting toxicity for irinotecan was observed. These results are of interest since anaemia has been reported in approximately 58.7% of patients receiving intravenous irinotecan monotherapy at the recommended dose and neutropenia and delayed diarrhoea are known to cause dose-limiting toxicity (33). These results confirm reports by Fiorentini *et al*(17) and Martin *et al*(32).

We report a relatively high incidence of post-procedural pain which is expected following hepatic arterial injection of irinotecan. Use of intravenous opioids and intra-arterial injection of 1% lidocaine has now become standard in the pain management protocols of patients undergoing DEBIRI treatment.

TACE is well established in the treatment algorithm for hepatocellular carcinoma and is becoming more accepted in the treatment of liver metastases from colorectal cancer. DEBIRI was found to be technically feasible in our study and since that time has gained increasing evidence as a viable treatment option for these patients. With respect to PK, average C_max_, AUC and t_½_ results for irinotecan in this study were 194 ng/ml, 1,680 ng•h/ml and 4.6 h, respectively. These results are considerably lower than reported values following i.v. administration of irinotecan (34) demonstrating the low bioavailability following DEBIRI treatment. In contrast, higher than expected levels of the main active metabolite SN-38 was observed. Average C_max_, AUC and t_½_ for SN-38 were 16.7 ng/ml, 281 ng•h/ml and 12.4 h, respectively. It is too early to conclude whether DEBIRI has any potential to increase the availability of SN-38 and further research is required to confirm this hypothesis.

Response rates in the current study showed stabilisation of the disease in 64% and a partial response in 18% of patients at 9 weeks after the first treatment. These response rates are in line with those reported in the second-line (refractory to 5-fluorouracil) phase II studies that formed the basis of approval for irinotecan monotherapy where response rates of 13.3–21.7% were achieved (35), however 9 weeks is a relatively short follow-up in this study and longer term data has now been published on the efficacy of DEBIRI. The time to progression of 154 days was shorter than previously reported but may reflect the patient population in which many had prior treatments for their colorectal disease. Following DEBIRI treatment, four patients were found to be eligible for laser-induced thermotherapy with the potential for better prognosis.

Our results for safety are consistent with recent publications on DEBIRI, but our efficacy results differ from recent reports. As this was the first clinical study of DEBIRI in mCRC we adopted the same technical approach for TACE in the treatment of hepatocellular carcinoma using a superselective stasis endpoint. Liver metastases from colorectal cancer are generally more hypovascular than HCC and may explain the lower than planned volume of DEBIRI delivered during the study. Since the time of our study, more experience has been gained on the optimised technique to use with DEBIRI. For patients with enough liver preserve, a more proximal catheter position and a lobar approach is now recommended to ensure delivery of DEBIRI to entire tumour area. The catheter should be positioned in lobar arteries past any extrahepatic vessel (e.g. cystic, pancreatic, gastroduodenal arteries). From there DEBIRI should be injected slowly (∼1 ml/min) until an endpoint of stasis in the intratumoral vessels whilst maintaining flow in the afferent vessels is achieved. It is not recommended to achieve stasis up to the catheter position. Our superselective approach with stasis endpoint may have missed some non-visible lesions causing them to be undertreated. This may explain the differences in efficacy results reported in our study compared to more recent publications.

Results from the present study are encouraging but are limited due to the small sample size and the relatively short follow-up. We demonstrated that DEBIRI therapy is a safe alternative treatment in the management of patients with hepatic metastases from colorectal cancer with an optimised pharmacokinetic profile as compared to systemic chemotherapy. Patients tolerated the treatment well at the doses and schedules used in this study which could allow this therapy to be considered in combination with systemic chemotherapy including irinotecan, oxaliplatin, as well as bevacizumab and cetuximab and other new biologic agents.

RECIST is a well established criteria for tumour response assessment in oncology trials, however, it is not appropriate for locoregional treatments such as DEBIRI. Assessment of tumour viability and tumour necrosis such as the mRECIST criteria in HCC (36) may provide a better assessment of treatment response. In our patients we were unable to measure tumour necrosis accurately. In addition, our technical approach was different to that reported in more recent publications and may explain the higher rate of progressions seen in our study. We are now participating in a German, multicentre, randomised study of DEBIRI + cetuximab (DEBIRITUX) vs irinotecan + cetuximab using the recommended DEBIRI technique (proximal approach) to assess progression-free survival of this combined treatment.

In conclusion, our study shows that chemoembolisation with irinotecan loaded DC Beads (DEBIRI) is safe, technically feasible and effective with a good PK profile. These positive and promising results have now been further investigated and confirmed in a larger patient population. Studies are underway to investigate this technology in all lines of therapy and in combination with existing systemic treatments for liver dominant disease from colorectal cancer.

## Figures and Tables

**Table I:** Patient demographics and tumour characteristics.

Demographics	n		%
Male/female	8/3		73/27
Caucasian/other	11/0		100/0
Age (mean ± SD, range)		64±12, 46–85	
ECOG 0/1	11/0		100/0
No of lesions			
1	5		46
2	4		36
3	1		9
>3	1		9
Sum of longest diameter, mm (mean ± SD, range)		63±25, 19–102	
Liver involvement, % (mean ± SD, range)		8.2±2.6, 4–12	

Prior chemotherapy (data from 8 patients): FOLFOX 6; 5-FU (2 cycles), oral Xeloda; CAPOX + FOLFOX, oxaliplatin/Tomudex, Tomudex monotherapy; 5-FU. No previous chemotherapy (3 patients): FOLFOX (6 cycles); FOLFOX (5 cycles); FOLFIRI (6 cycles), folinic acid + 5-FU + irinotecan + Avastin.

**Table II:** Adverse events.

System organ class	Preferred term	Events		Patients	
Cardiac disorders	Total	1	2.4%	1	9.1%
	Palpitations	1	2.4%	1	9.1%
Gastrointestinal disorders	Total	26	63.4%	9	81.8%
	Abdominal discomfort	2	4.9%	1	9.1%
	Abdominal pain	6	14.6%	5	45.5%
	Constipation	1	2.4%	1	9.1%
	Nausea	9	22.0%	6	54.5%
	Vomiting	8	19.5%	7	63.6%
General disorders and administration site conditions	Total	3	7.3%	3	27.3%
	Asthenia	1	2.4%	1	9.1%
	Pain	2	4.9%	2	18.1%
Infections and infestations	Total	1	2.4%	1	9.1%
	Device related infection	1	2.4%	1	9.1%
Investigations	Total	7	17.1%	2	18.1%
	Blood pressure increased	4	9.8%	1	9.1%
	White blood cell count increased	3	7.3%	1	9.1%
Musculoskeletal and connective tissue disorders	Total	1	2.4%	1	9.1%
	Musculoskeletal chest pain	1	2.4%	1	9.1%
Respiratory, thoracic and mediastinal disorders	Total	2	4.9%	2	18.1%
	Dyspnoea	2	4.9%	2	18.1%
Total		41		11	

**Table III:** Average pharmacokinetic parameters for irinotecan and SN-38.

	C_max_ (ng/ml)	T_max_ (h)	AUC_t_ (ng•h/ml)	AUC_24_ (ng•h/ml)	AUC (ng•h/ml)	λ_z_ (1/h)	t_1/2_ (h)
Irinotecan							
Mean	194	2^a^	1,510	1,520	1,680	0.1502	4.6^b^
SD	124		1,050	1,040	1,200	0.1346	
SN-38							
Mean	16.7	1^a^	147	147	281	0.0559	12.4^b^
SD	11.3		99	99	352	0.0238	

a Value is the median;

b calculated as ln2/mean λ_z_.

**Table IV:** Tumour response according to RECIST.

Patient	Post-treatment 1 (week 3)	Post-treatment 2 (week 6)	Post-treatment 3 (week 9)	Post-treatment 4 (week 12)	Follow-up (week 24)
001	Stable	Stable	Stable	Progression	-
002	Stable	Stable	Stable	Stable	Progression
003	Stable	Stable	Progression	-	-
004	Stable	Stable	Stable	Stable	-
005	Stable	Stable	Stable	Stable	Progression
006	Stable	Stable	Partial response	Partial response	-
007	Progression	Progression	-	-	-
008	Stable	Stable	Stable	Stable	-
009	Stable	Stable	Stable	Stable	Progression
010	Stable	Stable	Stable	Partial response	-
011	Progression	-	-	-	-
